# Optical Vapor Sensing on Single Wing Scales and on Whole Wings of the *Albulina metallica* Butterfly

**DOI:** 10.3390/s18124282

**Published:** 2018-12-05

**Authors:** Krisztián Kertész, Gábor Piszter, Zsolt Bálint, László Péter Biró

**Affiliations:** 1 Institute of Technical Physics and Materials Science, Centre for Energy Research, P.O. Box 49, H-1525 Budapest, Hungary; piszter@mfa.kfki.hu (G.P.); biro@mfa.kfki.hu (L.P.B.); 2 Hungarian Natural History Museum, Baross utca 13, H-1088 Budapest, Hungary; balint.zsolt@nhmus.hu

**Keywords:** butterfly wing, photonic crystal, chemically-selective vapor sensing, single scales, immersion, pigment content

## Abstract

Fast, chemically-selective sensing of vapors using an optical readout can be achieved with the photonic nanoarchitectures occurring in the wing scales of butterflies possessing structural color. These nanoarchitectures are built of chitin and air. The *Albulina metallica* butterfly is remarkable as both the dorsal (blue) and ventral (gold-green) cover scales are colored by the same type (pepper-pot) of photonic nanoarchitecture, exhibiting only a short-range order. The vapors of ten different volatiles were tested for sensing on whole wing pieces and some of the volatiles were tested on single scales as well, both in reflected and transmitted light. Chemically-selective responses were obtained showing that selectivity can be increased by using arrays of sensors. The sensing behavior is similar in single scales and on whole wing pieces, and is similar in reflected and transmitted light. By immersing single scales in an index-matching fluid for chitin, both the light scattering and the photonic nanoarchitecture were switched off, and the differences in pigment content were revealed. By artificially stacking several layers of blue scales on top of each other, both the intensity of the characteristic photonic signal in air and the magnitude of the vapor sensing response for 50% ethanol vapor in artificial air were increased.

## 1. Introduction

Natural photonic nanoarchitectures are valuable sources of information since they were optimized during many millennia of biological evolution [1,2]. These intricate structures, which generate structural colors, can be found in plants [3,4,5], birds [6,7,8,9], aquatic animals [10,11], and the most spectacular examples are in the insect world [12,13]. Photonic nanoarchitectures are nanocomposites that can interact with light in a spectrally selective way [13]. This unique property has inspired scientists to build devices with biomimetic approaches [13,14,15,16] and use these structures for various applications [17].

The photonic nanoarchitectures occurring in insect cuticles or bird feathers show an optical response when the vapor composition of the surrounding atmosphere changes [18,19,20]. This change of structural color is reversible: when the photonic nanoarchitecture is subjected to ambient air after the vapor exposure, the initial color is fully restored [18]. This makes them suitable for optical vapor sensing applications [21]. The vapor-induced changes of structural colors were investigated in many butterfly species with different photonic nanoarchitectures [20,22,23,24]. It was found that the porous three-dimensional photonic nanoarchitectures are the most attractive candidates for this task, as their open-air structure allows fast interaction with volatiles in the entire volume of the nanoarchitecture, which results in high vapor-induced color-change signals [18,24,25]. Based on these natural porous photonic nanoarchitectures, bioinspired structures for optical sensing were developed [17,26].

Recently, we investigated the vapor sensing properties of three-dimensional nanoporous “pepper-pot”-type nanoarchitectures occurring in the wing scales of polyommatinae blue butterflies [25]. It was found that the optical response of the wing, i.e., the wavelength shift of the reflected spectrum, is vapor specific. At lower concentrations, capillary condensation of the vapors into the nanoarchitecture was the governing process, while at high concentrations, changes of the chitin matrix (swelling) became dominant [25]. These advantageous properties of polyommatinae butterfly wings and the pepper-pot-type photonic nanoarchitectures in the lumen of the wing scales make them a promising model sensor material since, in addition to chemical sensitivity, the nanoarchitecture and generated structural color show high temporal and geographical stability [27]. Therefore, they can be considered as the first step towards the development of bioinspired artificial sensors based on photonic nanoarchitectures, can be used as biopolymer-based model sensors that are capable of chemically-selective sensing due to their special features (capillary condensation and swelling), and are available in macroscopic size. An additional advantage is that this type of photonic nanoarchitecture is characterized by a short-range order [28], i.e., no crystalline perfection is needed.

The *Albulina metallica* butterfly species is special, as the males have similarly colored dorsal wing surfaces as compared to the polyommatinae blues (e.g., *Polyommatus icarus*), while the ventral side has green coloration, also of structural origin [29]. In our earlier work, we showed that the vivid wing colorations of both sides are generated by quasi-ordered, pepper-pot-type photonic nanoarchitectures [29]. We showed, that despite the almost-identical scanning and cross-sectional transmission electron microscope images of the photonic nanoarchitectures, there are minor but characteristic differences between the two sides in respect to the size and distribution of the air holes filling the chitin matrix [30]. Direct space analysis (DSA) of the electron microscope images showed that the typical first-neighbor distance of the embedded air holes is characteristically different for the blue and green wing scales [30].

Most often the wings of butterflies are covered by several layers of scales, so that they are built up as follows, dorsal cover scales/dorsal ground scales/wing membrane/ventral ground scales/ventral cover scales [31,32]. Light and vapors interact with all of these layers [33]. To obtain more insight on the processes taking place when using entire wings as sensor materials, single-scale measurements were also carried out.

In this work, the vapor sensing properties of single scales and of whole wings for the two differently colored wing sides of the male *Albulina metallica* are reported. It was found that the dorsal (blue) and ventral (green) sides show characteristic optical responses to the test volatiles. Using principal component analysis (PCA), the vapor sensing spectra of the whole wing measurements were evaluated. We found that the structural colors of the two sides, and therefore the photonic nanoarchitectures generating them, have different vapor sensing properties. We show for the first time that separated single wing scales containing photonic nanoarchitectures can be used for optical vapor sensing. Using an optical microscope setup, the vapor sensing properties of the single blue and green scales were investigated. It was found that the optical responses are also vapor specific, similar to the measurements on the whole wings, and sensitivity to the ethanol test vapor of the single scales was enhanced by stacking several scales onto each other. These findings show that naturally tuned photonic nanoarchitectures can be used to construct sensor arrays consisting of whole wings, or consisting of one or a few scale stacks with different chemical selectivity, enabling the production of small (approximately 100 µm size) and material specific vapor sensors with optical readout.

## 2. Materials and Methods

The species *Albulina metallica* is not subjected to any restriction. The samples used in this work belong to the curated collection of the Hungarian Natural History Museum. For the whole wing optical spectroscopy measurements, we used the Avantes (Avantes BV, Apeldoorn, The Netherlands) modular fiberoptic system (for details, see a past paper [34]). All chemicals used for the vapor sensing experiments are “reagent grade” class and were obtained from VWR (VWR International Ltd., Radnor, PA, USA). Both dorsal and ventral wing sides were used to measure vapor response, twice, one after another, and on consecutive days while turning the sides. The entire measurement was performed twice by two operators with complete disassembly of the setup between the two runs to avoid systematic errors. The relatively large size of the wing (>1 cm) and its complex relief pattern lead to a new incident and reflected light angle with every new setup, but the angles were tuned every time to obtain the highest possible reflection. All other experimental parameters were kept the same during the eight measurements. The temperature in the laboratory was kept constant (24 °C), and a thermally stabilized Peltier element was placed under the wing sample and set to a constant 18 °C. The Peltier temperature setting and the gas-flow switching protocols were controlled from LabView with a predefined flowchart.

Reflected and transmitted light optical microscopy were carried out using a Zeiss AxioImage A1 (Carl Zeiss AG, Jena, Germany). The index-matching oil was Zeiss Immersol 518 F. For microspectrometry, the light was collected through an adapter tube attached to the upper port of the microscope and conducted with an optical fiber to the Avantes (HS1024 × 122TEC) spectrometer.

Scanning electron microscopy images were taken using an LEO 1540 XB (Carl Zeiss AG, Jena, Germany) microscope on wing pieces attached with conductive tape without any preparation.

We carried out all data processing (including the PCA) and representation using Origin 2018 software (OriginLab Co., Northampton, MA, USA).

## 3. Results

**Figure 1:**
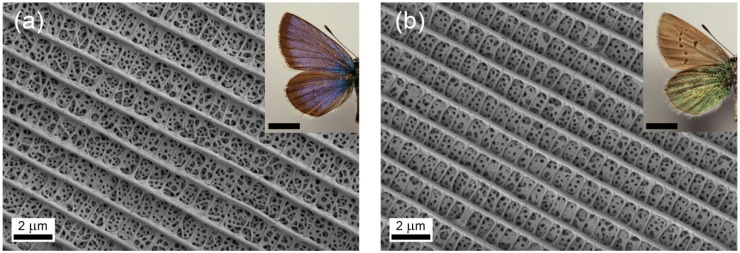
Scanning electron micrographs of (**a**) blue and (**b**) gold-green scales (imagine photos as inset, scale bar: 5 mm) of *Albulina metallica* male specimen.

To compare the two sides of the butterfly wings, we investigated wing pieces under a scanning electron microscope. In the wing scales, similar pepper-pot-type photonic nanoarchitectures were observed (Figure 1). The color insets show the corresponding dorsal and ventral wing surfaces of the imagines. Despite the similarity in the quasi-ordered nanoarchitectures, the measured spectral differences were significant, as the characteristic reflectance peaks appeared at 420 nm on the blue side and at 560 nm on the green side [29]. As we showed earlier, the structural differences of the two sides of *Albulina metallica* males can be revealed by DSA image processing of the electron micrographs [30] based on the characteristic differences in the first-neighbor distance between the air voids in the nanoarchitectures.

**Figure 2:**
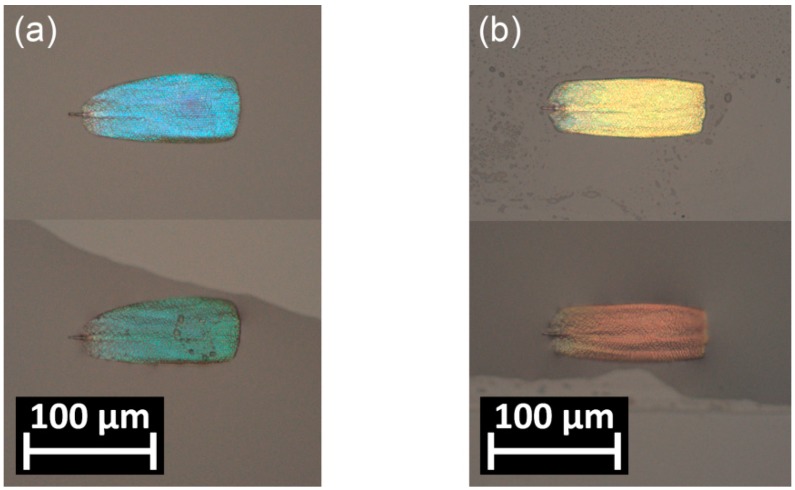
Reflected light optical micrographs of *Albulina metallica* single scales in air (upper) and submerged in ethanol (lower): (**a**) blue and (**b**) gold-green scales. The color changes from blue to green and from gold-green to red, respectively.

The reflectance spectrum is determined by the periodicity and the refractive index contrast of the materials building up the nanoarchitecture. A relatively high spectral shift occurs when the air voids in the chitinous matrix are filled with a liquid with different refractive index than the initial (air). To present this effect, single separated wing scales from the dorsal and ventral sides of the male specimen were placed on a microscope slide. Figure 2 shows the color differences of the wing scales in ambient air, and when they were submerged in liquid ethanol.

**Figure 3:**
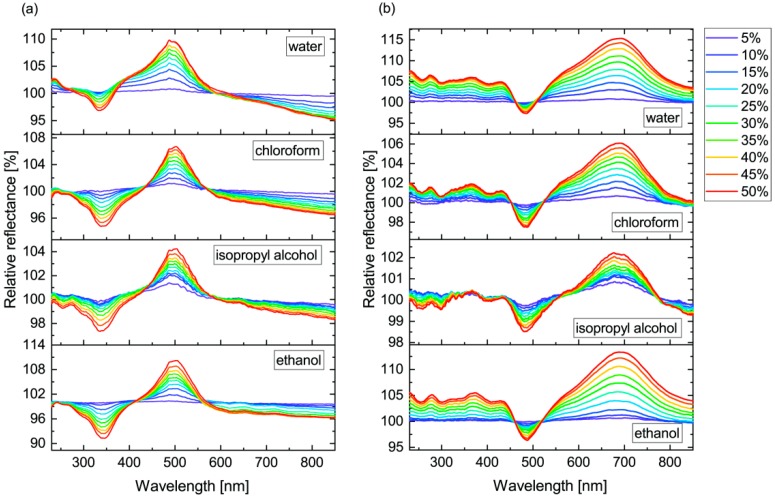
Averaged relative reflectance spectra of water, chloroform, isopropyl alcohol, and ethanol measured on the (**a**) blue and (**b**) gold-green sides of the *Albulina metallica* wing.

During whole wing vapor exposure measurements, the optical response for ten vapors were measured at increasing concentrations from 0% to 50%, in 5% steps. A total of eight measurements were conducted: the two operators measured the vapor sensing properties of the two sides of the butterfly wing independently, each measurement was repeated twice. To facilitate the presentation and the interpretation of the measured data, the relative reflectance spectra were introduced [18] using the initial spectra of the wings in artificial air ($R_{0}$) as a reference: $\Delta R=\left(R/R_{0}\right)\times 100\%$. The relative reflectance spectra are shown at every vapor concentration step in Figure 3.

**Figure 4:**
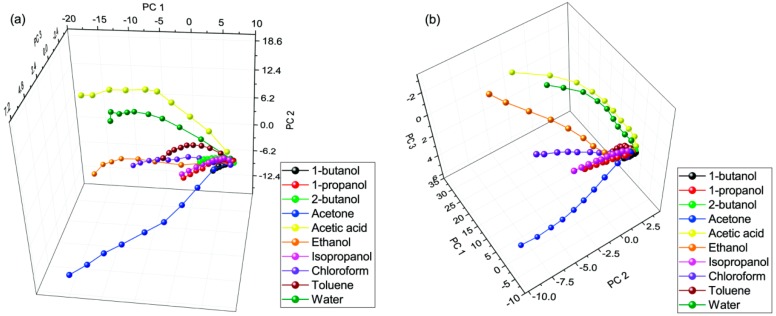
Principal component analysis (PCA) scores of the first three principal components (PCs) of the vapor sensing datasets measured on (**a**) blue and (**b**) gold-green *Albulina metallica* wing surfaces. The radially spreading paths from a common origin correspond to increasing concentrations of the vapors. The axes were rotated to facilitate the comparison.

On both wing surfaces, we carried out a total number of four measurements that were averaged to follow the characteristic evolution of the response signal. As an example, we show the averaged response signal from 0% to 50% in the case of the vapors of chemically different compounds: water, chloroform, isopropyl alcohol, and ethanol (Figure 3). To compare the chemical selectivity for all applied vapors, principal component analysis of the measured data was used. For this purpose, the measured spectra were cropped to 230–850 nm, where the spectral shift originating from the photonic nanoarchitecture occurred. The PCA scores of the vapor sensing measurements are plotted in Figure 4 for both wing surfaces. We observed >96% cumulative variance for the first three principal components (PCs).

**Figure 5:**
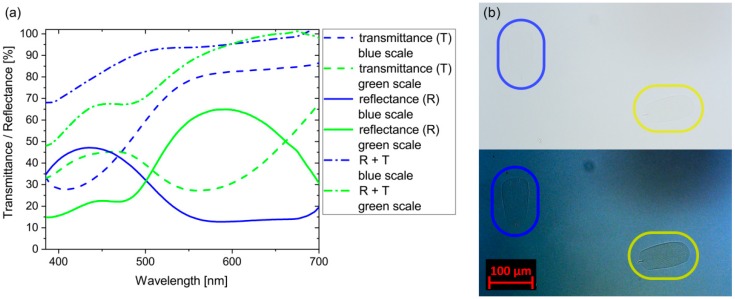
(**a**) Reflectance and transmittance measurements on single blue and gold-green *Albulina metallica* scales and (**b**) micrograph of single scales immersed in index-matching oil. The dorsal scale is highlighted with blue, the ventral with yellow. The upper panel is the original image, the lower panel is contrast enhanced for better visibility.

To obtain insight in the processes taking place on single scales, we separated and placed them on glass microscope slides to carry out reflectance and transmittance measurements. For reference, we used the light reflected from and transmitted through the glass slide. In Figure 5a, continuous lines represent the reflectance spectra of the single blue and gold-green scales, while the dashed lines show the transmittance measured at the same spot without manipulation of any mechanical part of the microscope; only the direction of the illumination was changed. Dash-dot lines were calculated as the sum of the reflected and transmitted spectra for blue and gold-green wing scales. Figure 5b shows the image of the wing scales in transmitted light when they were immersed in an index-matching oil. The contrast was enhanced for better visibility. The transmittance of the immersed blue and gold-green scales was measured to be 99% and 95% at 450 nm, respectively.

**Figure 6:**
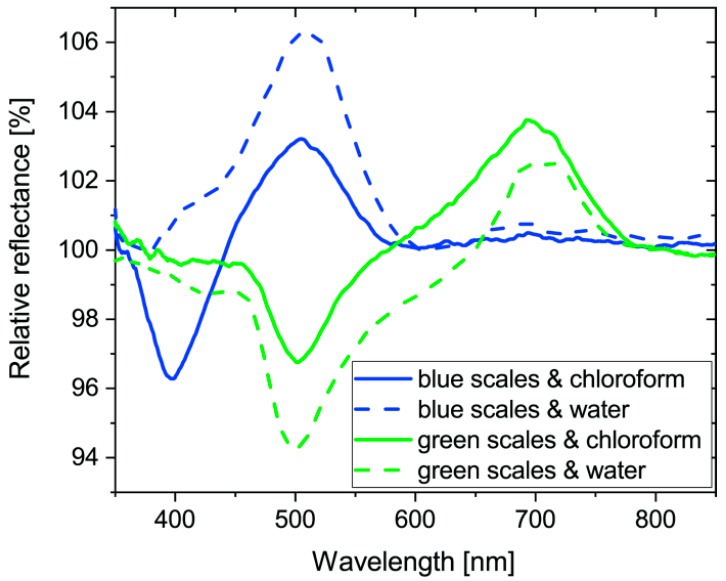
Average of vapor sensing signals (in reflected light) on single blue and gold-green *Albulina metallica* wing scales using 50% chloroform and water vapors, as noted. Measurements were conducted on single scales still attached to the wing membrane.

After the whole wing vapor sensing measurements, single scale measurements were carried out. The butterfly wing in the gas cell was investigated using an optical microscope. The 20× objective had enough working distance to be able to focus on single scales inside the cell. We performed vapor sensing measurements on ten blue and ten gold-green wing scales while applying a 50% concentration of chloroform and water vapors. The average of the ten measurements on each side and for each vapor is plotted in Figure 6. Please note the wavelength range of the graph is narrower than in the whole wing measurements, because of the restricted transmittance of the glass optical elements in the microscope.

**Figure 7:**
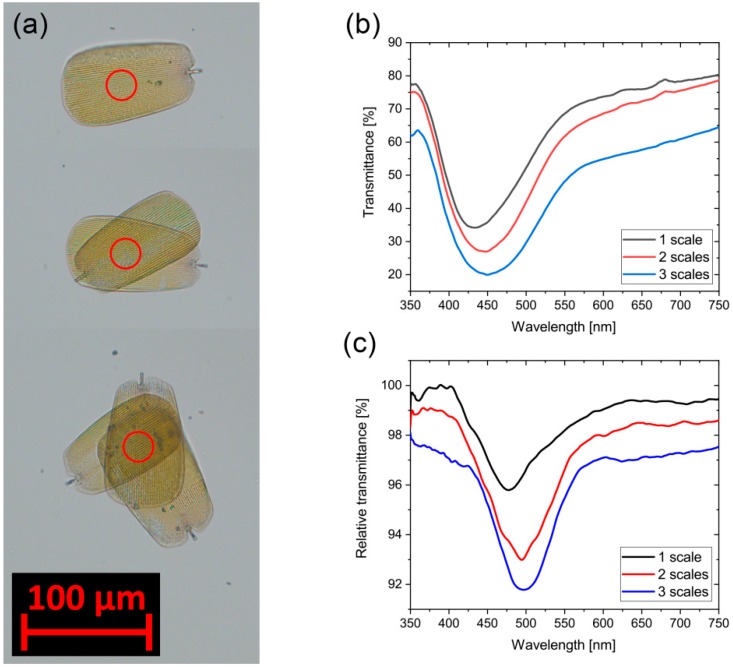
(**a**) Micrograph of stacked dorsal *Albulina metallica* scales in transmitted light; transmittance was measured in the center of the red circles; (**b**) transmittance spectra measured at the overlapping center; and (**c**) vapor sensing signals measured at the same spot using 50% ethanol (ten point adjacent-average filtered curves) in transmitted light.

The effect of stacked wing scales on the vapor-sensing properties was also investigated. Individual blue scales were placed on top of each other on a microscope cover slide (to have as small as possible an influence on the light path) using a sharp needle. In this setup, we measured transmittance of light across one, two, and three stacked scales. Figure 7a shows the optical images in transmitted light. Using the light passing through the cover slide as a reference, we measured the transmittance of one, two, and three layers of scales (Figure 7b); we also measured their optical response in transmitted light by applying 50% ethanol vapor (Figure 7c). Similar to the measurements in reflected light carried out on the whole wings, the relative transmittance here is defined as the quotient of transmission under 50% ethanol vapor divided by transmission in air.

## 4. Discussion

Structural coloration of both the dorsal and ventral wing surfaces is not a very frequent phenomenon for butterflies possessing structural coloration [28,35]. Moreover, in the case of *A. metallica*, the morphology of the wing scales on the dorsal and ventral sides is very similar; the internal nanoarchitectures are hard to distinguish by only qualitative analysis of the scanning electron microscope (SEM) (Figure 1) and transmission electron microscope (TEM) images [28]. Therefore, this butterfly offers an attractive opportunity to investigate the effects arising from moderate differences in the vapor sensing of photonic nanoarchitectures exhibiting only a short-range order.

Photonic crystals, understood in a strict sense, suppose a well-ordered structure; however, as was demonstrated by theoretical modeling, disordering the structure while preserving its periodicity does not destroy the photonic band gap [36]. In our case, the first neighbor distance follows a certain distribution for both the structurally colored scales on the dorsal (blue) and the ventral (green) wing sides [30], and this enables two different reflectance maxima positions, therefore the different dorsal and ventral wing colors (Figure 1).

The color of the scales is given by the particular nanostructure and refractive index contrast of the building materials. We obtained a considerable color change on single scales from the dorsal and ventral sides of the wings by immersing them in liquid ethanol under an optical microscope (Figure 2). The colors were shifted toward the longer wavelengths: blue to green, and gold-green to red. This process could be repeated without damage to the scales; after a short drying period (order of seconds), the color was fully recovered. As we showed earlier, a similar process, based on the cold condensation of atmospheric humidity in the nanopores of the scales, enables “writing” on structurally colored scales by the local cooling of the scales [37]. Smaller magnitude but similar change occurs while replacing the ambient air with a vapor + air mixture from which capillary condensation takes place [38].

In Figure 3, the characteristic differences for four different vapors are shown as an example. One may observe that these different chemical compounds generate characteristic sets of curves, both for the chemical compound—chemical selectivity—and for the particular photonic nanoarchitecture—structural specificity—all of which show concentration dependence. Despite the apparent similarity in the wing-scale structures, the scales on the two wing sides present different optical responses. One may conclude that even minor differences in the parameters of the nanoarchitectures are sufficient to determine a specific response. This shows that, by combining different nanoarchitectures in an array of sensors, selectivity can be enhanced by using the previous “fingerprinting” of various vapors and storing the characteristic responses on the different elements of the array.

Based on the first report on chemical sensing of photonic nanoarchitectures occurring in butterfly scales [18], chemical selectivity is demonstrated by performing principal component analysis (PCA), using the easily readable 3D score plots. When using the same method on the same plot to evaluate the changes induced by all ten vapors on both wing sides, while the axis orientation and scales are different, the shape and ordering of the curves look similar (Figure 4). The different orientation of the axes in the 3D PCA curves underlines the differences of the responses of the two similar, but different, nanoarchitectures. It is important to note that the input data for the PCA on the two wing sides are different in terms of where the maximum and minimum wavelengths appear in the spectra; still, the 3D plots look very similar after a proper graph rotation. This shows that despite the characteristic differences, the processes inducing the modification of the optical response are essentially similar for the scales on the blue and green wing sides. Longer paths, meaning higher sensitivity, occur for acetone, acetic acid, ethanol, chloroform, and water. There is lower sensitivity for 1-butanol, 1-propanol, 2-butanol, isopropyl alcohol, and toluene. The similar characteristics are explained by the qualitatively identical scale nanostructure, while the differences are attributed to the quantitative features, which are also responsible for the different colors generated.

The sensitivity on the whole wing is the cumulative effect of the single scales. One scale has a length and width of ca. 100 by 50 μm; an optical microscope is needed for the inspection of individual scales. We observed complementary spectral behavior in the reflected and transmitted light on a single cover scale detached from any of the two sides of the wing (Figure 5a); the blue continuous vs. blue dashed lines and green continuous vs. green dashed lines are, to a good extent, complementary to each other. This agrees with our first expectation to a selective reflector. The wavelengths falling in the reflected region must be reduced or absent in the transmitted light and vice versa. The sums of the reflected and transmitted light intensities are plotted with dash-dot lines in Figure 5a. There is a difference of the sum from the 100% plateau increasing towards the shorter wavelengths. The scales are layered chitinous nanomaterials reflecting the incident light according to their photonic crystal-type behavior and transmitting another significant part. Furthermore, they can absorb light, if they contain a pigment, or scatter the light. By switching off the photonic crystal, immersing the scale in an oil with refractive index *n* = 1.518, close to the refractive index of the chitin, one can observe in transmitted light the contribution of the absorption (Figure 5b). While the scales are in immersion, both the reflection due to the photonic crystal and the scattering are switched off. On the optical micrographs, there is a hardly visible outline of the two scales; however, the gold-green scale (yellow encircling) is more visible. After contrast enhancement, a clearly visible difference appears between the two scales, showing that the blue one contains less pigment. This result agrees with Figure 5a, where the blue dashed-dot line is closer to 100% indicating less absorbing material in the blue scale. For both types of scales, the missing fraction of the light towards short wavelengths is attributed to light scattering and the absorption of the pigments, as the gold-green scale contains more pigment, more light is missing in the short wavelength side.

One of the most important advantages of the photonic sensors consists in the possibility of miniaturization. As the whole wing can be considered as a mosaic surface of numerous scales, one scale must show similar properties to the wing itself. We performed vapor sensing measurements placing the gas cell under the microscope objective and focusing onto the middle of scales. The average of difference signals on ten scales is shown in Figure 6 from both sides using water and chloroform vapors. The concentration was set to 50%, since we were not targeting to survey the complete sensitivity curves, rather just show the main effect. It seems that it is worth further investigation on a small sensing area. The signals show similar characteristics with the whole wing measurement even if the optical microscope has a rather attenuated transmittance outside the 400 to 700 nm (human-visible) range.

In the macroscopic setup where a ~5 × 5 mm^2^ wing piece was placed in the gas-sensing cell only reflected light can be used to detect the variations due to the multiple scale layers both on the dorsal and ventral sides. On the other hand, single scales placed on flat and transparent support (glass slide in our case) enable transmittance measurement. Such an arrangement may be more advantageous for the construction of miniaturized sensors. As we presented before, the transmitted spectra are nearly complementary to the reflected ones, therefore one expects a similar sensing behavior in transmittance, too. Furthermore, such an arrangement allows for the increase of the signal by stacking several scales on top of each other. Here, we placed single dorsal blue scales on top of each other (Figure 7a) and measured the transmittance at the central position of the overlapping areas. According to the micrographs, the overlapping scales look darker and the transmittance spectra (Figure 7b) showed decreasing intensities with a peak (gap) at 450 nm. Exactly the same location where the transmittance was measured, we recorded vapor sensing signals for 50% ethanol vapors. On the wings, there are commonly two or more layers of scales, but mostly only the cover scales contain a photonic nanostructure. In this measurement with the increasing signals, we demonstrated the increase in sensitivity with the increase of the volume of interaction between vapors (and condensed vapors) and nanoarchitectures.

## 5. Conclusions

Selective chemical sensing of two qualitatively similar, but quantitatively different, “pepper-pot”-type photonic nanoarchitectures, which occur in the dorsal (blue) and ventral (gold-green) cover scales in the wings of the male *Albulina metallica* butterflies, were investigated using ten different vapors. Complete immersion of single scales in ethanol showed marked changes from blue to green and from gold-green to red. The capillary condensation of four vapors: water, chloroform, isopropyl alcohol, and ethanol, yielded characteristic response curves in relative reflectance both for the chemical substances and for the two different structures in the concentration range of 5% to 50% vapor in artificial air. The trajectories obtained by principal component analysis for ten different vapors in the same concentration range revealed quantitatively different but qualitatively similar responses for the two nanoarchitectures.

As the complete wings are optically complex objects, we also investigated the behavior of single scales, both by using a microscope and collecting the signals not from the entire wing, but from one single scale only, by removing single scales from the wing and placing them on microscope slides for individual characterization. We found that the reflected and transmitted light were complementary to a good extent, but towards the short wavelength side an increasing amount of light was missing. It was found that, in the case of the gold-green scales, more light was missing as compared to the blue scales. This is attributed to the higher concentration of pigment present in the gold-green scales. In both types of scales, light scattering also contributes to the loss in intensity of the reflected and transmitted beams. The single scales exhibit similar chemically-selective sensing both in reflected light and transmitted light as was found on the whole wings. By artificially stacking several layers of scales possessing photonic nanoarchitectures, we demonstrated that the magnitude of the sensing signal can be increased.
